# Methylphenidate in mania project (MEMAP): study protocol of an international randomised double-blind placebo-controlled study on the initial treatment of acute mania with methylphenidate

**DOI:** 10.1186/1471-244X-13-71

**Published:** 2013-02-27

**Authors:** Michael Kluge, Ulrich Hegerl, Christian Sander, Jens Dietzel, Roland Mergl, Istvan Bitter, Koen Demyttenaere, Ricardo Gusmão, Ana Gonzalez-Pinto, Victor Perez-Sola, Eduard Vieta, Georg Juckel, Ulrich S Zimmermann, Michael Bauer, Pascal Sienaert, Sónia Quintão, Marc-Andreas Edel, Csilla Bolyos, Jose Luis Ayuso-Mateos, Pilar López-García

**Affiliations:** 1 Department of Psychiatry and Psychotherapy, University of Leipzig, Semmelweisstrasse 10, 04103, Leipzig, Germany; 2 Department of Psychiatry and Psychotherapy, Semmelweis University, Budapest, Hungary; 3 Department of Psychiatry, University Psychiatric Center KU Leuven, Leuven, Belgium; 4 CEDOC, Clínica Universitária de Psiquiatria e Saúde Mental, Faculdade de Ciências Médicas de Lisboa, Lisbon, Portugal; 5 Department of Psychiatry, Hospital Santiago Apostol, Vitoria, Spain; 6 Centro de Investigació n Biomé dica en Red de Salud Mental. CIBERSAM, Madrid, Spain; 7 Department of Psychiatry, Hospital de la Santa Creu i Sant Pau, Barcelona, Spain; 8 Department of Psychiatry, Hospital Clinic, University of Barcelona, IDIBAPS, Barcelona, Spain; 9 Department of Psychiatry and Psychotherapy, University of Bochum, Bochum, Germany; 10 Department of Psychiatry and Psychotherapy, University Hospital Dresden, Dresden, Germany; 11 Department of Psychiatry, Universidad Autonoma de Madrid, Madrid, Spain; 12 Instituto de Investigación Sanitaria Princesa (IP), Madrid, Spain

**Keywords:** Psychostimulants, Mania, Bipolar disorder, Methylphenidate, Vigilance, EEG

## Abstract

**Background:**

Treatment of patients with acute mania remains a considerable medical challenge since onset of action of antimanic medication is delayed for several days. Psychostimulants could have an earlier onset of action. This assumption is based on the ‘vigilance regulation model of mania’ which postulates that vigilance is unstable in manic patients. Accordingly, vigilance-stabilising psychostimulants could be more useful than conventional treatment in acute mania. We present here the study protocol of a trial intended to study the efficacy and safety of methylphenidate in the initial treatment of acute mania.

**Methods/design:**

A multi-centre, randomised, double-blind, placebo-controlled clinical trial will be conducted in 88 bipolar inpatients with acute mania. Male and female patients older than 18 years will be randomised to treatment with either methylphenidate (20 to 40 mg/day) or placebo for 2.5 days, given once or twice daily. The main outcome measure is the reduction in the Young Mania Rating Scale (YMRS) after 2.5 days of treatment. Other outcome measures include the Positive and Negative Syndrome Scale-Excited Component (PANSS-EC) the Clinical Global Impression–Bipolar Scale (CGI-BP), the Screen for Cognitive Impairment in Psychiatry (SCIP), actigraphy and the EEG-‘Vigilance Algorithm Leipzig’ (VIGALL).

**Discussion:**

A positive study outcome of the proposed study could substantially impact our understanding of the etiopathogenesis of mania and open new treatment perspectives.

**Trial registration:**

ClinicalTrials.gov: NCT 01541605

## Background

Treatment of patients with acute mania remains a considerable medical challenge, amongst other reasons because manic patients often lack insight into their illness and into the necessity being treated. Therefore, there is a need for rapidly acting antimanic substances since initial willingness being treated may change and then seriously compromise treatment outcome. Yet, onset of action of medication commonly used in the treatment of mania is delayed for at least several days (e. g. haloperidol, lorazepam, olanzapine, risperidone, valproate) or more (e. g. lithium, carbamazepine) [1-3]. Faster onset of action of antimanic medication would not only shorten the burden of patients but also significantly decrease cost due to shorter duration of hospital stay [1].

**Figure 1:**
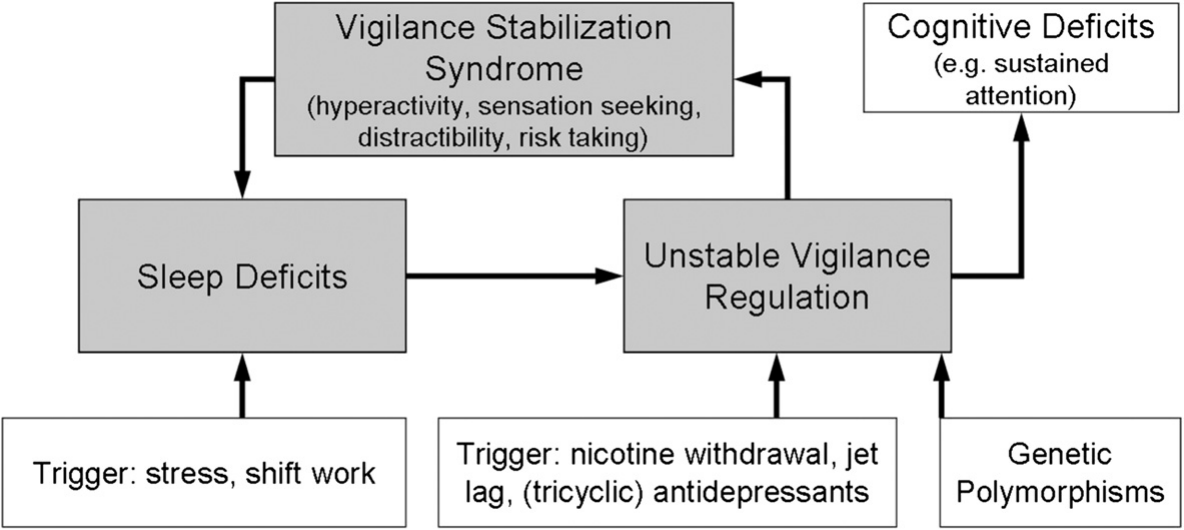
**The vigilance regulation model of mania: Unstable vigilance induces a pathogenic circle with vigilance stabilisation syndrome leading to full-blown mania.** Figure taken from: Hegerl U., et al. Are psychostimulants a treatment option in mania? Pharmacopsychiatry 2009; 42:169-174. Reproduced with kind permission of Georg Thieme Verlag KG”.

While above mentioned substances are sedating, we intend to use a rapidly acting, *stimulating* substance in this study. This plan is based on the ‘vigilance regulation model of mania’. It takes into account that the vigilance level –vigilance defined as brain arousal– does not only influence behaviour but that in turn, behaviour can also affect the level of vigilance; i.e. a more/less stimulating environment can be actively created in order to increase/reduce vigilance levels. For example, overtired children often develop a hyperactive, talkative and sensation seeking behaviour which can be interpreted as an autoregulatory attempt to stabilise vigilance by increasing external stimulation. We postulate that this physiological autoregulatory mechanism may result in a pathological behavioural syndrome, namely mania, in vulnerable subjects [4-6]. The autoregulatory mechanism might override the physiological tendency to seek sleep, thus aggravating sleep deficits, worsening vigilance instability and thereby starting a vicious circle ending up in full-blown mania (Figure 1) [4].

The outlined model has already been proposed earlier [7,8] and is related to personality theories about extraversion [9] and sensation seeking [10] which comparably explain these traits as an attempt to compensate for low central nervous system arousal.

The ‘vigilance regulation model of mania’ provides an explanation for several seemingly paradoxical aspects and is supported by several lines of evidence:

• In contrast to the clinical observation of hyperactivity, there is robust evidence from EEG recordings that vigilance is unstable in manic patients: under quiet rest, manic patients show rapid decline to low vigilance stages often already within the first seconds of EEG recording [4]. In line with this, micro sleeps (abrupt intrusion of sleep spindles) were observed shortly after starting recordings [11,12].

• Sleep deficits can trigger or worsen manic behaviour [13,14] and life events disturbing sleep-wake-regulation can trigger or aggravate (hypo)manic syndromes [15]. Accordingly, stabilisation of sleep-wake rhythm is used in behavioural therapies for bipolar disorder [13,16].

• Withdrawal of vigilance-stabilising drugs, such as nicotine, can trigger mania [17,18] and a high smoking prevalence has been reported in bipolar disorder [19]. This may indicate that these patients may benefit from the vigilance-stabilising properties of nicotine. Consistently, smoking and coffee drinking were not associated with higher incidence of mania in bipolar disorder, when confounding factors were controlled for [20].

• In ADHD, a disease with high comorbidity and a broad symptom overlap with mania [21,22], psychostimulants effectively reduce attention deficits, sensation seeking behaviour and hyperactivity, symptoms which are also present in mania [23,24]. In addition, methylphenidate has been shown to improve sleep in children [25,26] and adult patients [27] with ADHD. Furthermore, randomised controlled trials in pediatric patients with ADHD and additional manic symptoms showed that psychostimulants are effective in reducing both ADHD and manic symptoms [28,29].

• There is increasing evidence that psychostimulants are effective in treating mania and that this effect is related to stabilisation of vigilance. A variety of case reports describe a pronounced and rapid improvement of manic symptoms after administration of psychostimulants in adult manic patients with bipolar disorder [4,30-33]. Bschor et al. [34] report on a manic patient with decreased vigilance who showed both a rapid (within 2 hours) and strong improvement of manic symptoms and a stabilisation of vigilance as assed in the EEG after administration of methylphenidate.

• Good response to the psychostimulant modafinil was recently described in a case report. Clinical improvement was observed already after one hour and it was associated with a stabilisation of vigilance [35]. In line with our model, psychostimulants were mostly not efficacious in the treatment of typical depression, except for apathy [36,37].

While psychostimulants have occasionally been linked to induction of mania [38] switches into mania occur rarely: A large evaluation by the U.S. Food and Drug Administration found a psychotic or manic-like reaction only in about 1 of 400 treated patients with ADHD and in the majority of cases (> 90%) the symptoms resolved within 2 days. Thus, the risk of an aggravation of manic symptoms appears to be low [39].

Onset of action of methylphenidate is fast, i.e. faster than 1 hour and maximum effects will be reached after approximately 2 hours [40]. Thus, methylphenidate is likely to provide a much faster effect on manic symptoms than other drugs commonly used in mania.

Taken together, there is increasing evidence from both case studies in adult manic patients and randomised controlled trials in pediatric patients with ADHD and concomitant manic symptoms that psychostimulants such as methylphenidate are effective in the rapid treatment of mania.

Aim of this study, the ‘Methylphenidate in Mania project’ (MEMAP), is to test for the first time the hypothesis that the psychostimulant methylphenidate has antimanic properties after 2.5 days of treatment using a double-blind placebo-controlled design.

### Research objectives

#### Primary objective

The primary objective of the study is to test the hypothesis that methylphenidate immediate release given twice daily (BID) is significantly superior to placebo in the treatment of manic symptoms in patients with bipolar disorder after 2.5 days of treatment as assessed by the Young Mania Rating Scale (YMRS) [41].

### Secondary objectives

Secondary objectives of this study comprise to evaluate

• Whether methylphenidate immediate release given BID is significantly superior to placebo in the treatment of manic symptoms in patients with bipolar disorder after 2 hours of treatment as assessed by the YMRS and the Positive and Negative Syndrome scale - excited component (PANSS-EC) [42].

• The change from baseline to endpoint (after 2.5 days of treatment) on the Clinical Global Impression-Bipolar Scale CGI-BP [43] and the PANSS-EC.

• Whether 2.5 days of treatment with methylphenidate but not with placebo stabilise vigilance regulation as assessed by the ‘Vigilance Algorithm Leipzig’ (VIGALL) [44].

Whether instability of vigilance regulation as assessed by the VIGALL predicts response to methylphenidate.

• Whether methylphenidate immediate release given BID is associated with significantly less movements over the study period than placebo as assessed by actigraphy.

• Whether methylphenidate is significantly superior to placebo in improving cognitive performance as assessed by the “Screen for Cognitive Impairment in Psychiatry” (SCIP) [45].

## Methods and design

### Design

MEMAP is a two-arm, randomised, placebo-controlled, double-blind, parallel, multi-centre phase IIIb exploratory study to evaluate the efficacy and safety of methylphenidate in the initial treatment of acute mania in patients with bipolar affective disorders. After being screened for eligibility and signing the informed consent (day -2 or day -1), patients will be randomised to either 2.5 day treatment with methylphenidate BID or placebo BID at a 1:1 ratio (day -1/day 0). Medication will be administered at 10.00 and 15.00 h on day 0 (15 mg methylphenidate or placebo BID), at 09.00 and 15.00 h on day 1 (20 mg methylphenidate or placebo BID), and at 09.00 h on day 2 (20 mg methylphenidate or placebo). EEG should be conducted prior to first medication and 2 h after the morning dose on day 2. Manic symptoms will be assessed using the YMRS. YMRS will be scored at baseline and 2 h after the morning dose on every day. In addition, the CGI-BP and PANSS-EC will be scored. Furthermore, movements will be recorded using actigraphy and a cognitive test (SCIP) [45] will be performed. The schedule of study assessments is described in detail in Table 1.

**Table 1:** Schedule of study assessments

**Examination/evaluation**	**Screening**	**Treatment**			**Follow up**	
**Day**	**-2/-1**	**0**	**1**	**2**	**3**	**9**
Review Inclusion/Exclusion criteria	**X**					
Informed consent	**X**					
Randomisation	*X	**(X)**				
**General**						
Medical and psychiatric history	**X**					
Demographics and illness characteristics	**X**					
Physical examination	**X**					
Vital signs	**X**	**X**	**X**	**X**	**X**	**X**
Blood analysis	**X**				**X**	**X**
Urine analysis, drug screening	**X**					**X**
Pregnancy test	*X					
Concomitant medication	**X**	**X**	**X**	**X**		**X**
Baseline findings/adverse events	**X**	**X**	**X**	**X**		**X**
ECG	**X**			**X**		**X**
ADHD-Screening					**X**	
**Efficacy**						
YMRS	**X**	**XX**	**X**	**X**	**X**	**X**
CGI-BP		**X**		**X**	**X**	**X**
PANSS-EC		**XXXXX**	**X**	**X**	**X**	**X**
EEG		**X**		**X**		
Actigraphy	*X	**X**	**X**	**X**		
Cognitive test		**X**	**X**	**X**		
**Medication**						
Study drug: methylphenidate, placebo		X*X*	X**X**	X**		
Up to 3 mg/day lorazepam or alprazolam	**X**					
Up to 2 mood-stabilisers allowed	**X**	**X**	**X**	**X**	**X**	**X**

*day -1 after informed consent.

Number of ‘X’ represents number of assessments (X* 15 mg methylphenidate vs. placebo, X** 20 mg methylphenidate vs. placebo).

Pre-existing medication for treatment of bipolar disorder that has been stable for at least 4 weeks including benzodiazepines, lithium, anticonvulsants or antipsychotics will be continued in the same dose. Only patients receiving up to 2 such drugs are eligible for study participation. During screening, the (additional) use of up to 3 mg/day lorazepam or alprazolam is allowed. The treatment period is 2.5 days. There will be two post treatment visits at day 3 and 9.

### Ethics

This international study will be conducted in Belgium, Germany, Hungary and Spain. Study sponsor is the University of Leipzig, Germany. The study protocol has been already approved by both the leading Ethics Committees (EC) and Health Authorities in Germany, Hungary and Spain as well as the responsible Health Authority in Belgium. The investigator is responsible for ensuring that no patient is subject to any study-related examination or activity before that patient has given written informed consent, after the receipt of detailed information. The investigator will inform the patient of the aims, methods, anticipated benefits and potential hazards of the study including any discomfort it may entail. This information will be summarized in integrated patient information and consent sheets. The study has been registered on the EudraCT database (EudraCT number 2010-023992-24) and at ClinicalTrials.gov (NCT01541605). The trial will be conducted in accordance with the latest version of the Declaration of Helsinki and the International Conference on Harmonization (ICH) - Good Clinical Practice (GCP) guidelines for clinical trials.

### Study population

The study population will comprise 88 adults of either sex with the diagnosis of a manic episode according to ICD-10, 44 in each arm of the study. To be included in the study, patients must fulfil all inclusion criteria and not meet an exclusion criterion.

**Inclusion criteria** are: 1. inpatients; 2. written informed consent by patients who are competent to consent to study participation; 3. diagnosis of a manic episode according to ICD-10 classification (F30.0, F30.1, F31.0 or F31.1); 4. male or female ≥ 18 years of age; 5. YMRS total score ≥ 20 and ≤ 45 points; 6. body mass index (BMI) > 17 and 7. patients must be able to swallow tablets (study drug).

**Exclusion criteria** are: 1. any other current major psychiatric ICD-10 disorder except for attention deficit hyperactivity disorder (ADHD) or other hyperkinetic disorders (F90), harmful use of tobacco (F17.1), dependence syndrome of tobacco (F17.2), neurotic, stress-related and somatoform disorders (F40-48), behavioural syndromes associated with physiological disturbances and physical factors (F50-F59) or disorders of adult personality and behaviour (F60-F69); 2. contraindications for treatment with methylphenidate except as noted otherwise including hypersensitivity to methylphenidate or components of the drug, patients with extreme anxiety and agitation, women with child bearing potency without effective contraception (i. e. implants, injectables, combined oral contraceptives, some IUDs or vasectomised partner) during the conduct of the trial. Patients using hormonal methods of contraception must be informed about possible influences of the study drug on contraception; 3. serious non-psychiatric disease (e.g. infectious, autoimmune or metabolic), that may interfere with the objectives of the study or with the safety or compliance of the subject, as judged by the investigator; 4. oral administration of MAO-inhibitors within two weeks, fluoxetine within 6 weeks and of any other antidepressant or primarily psychotropic substance except for those specified below within one week before study entry; 5. stable treatment with mood stabilisers including lithium, anticonvulsants (e.g. valproate, carbamazepine) or antipsychotics (e.g. risperidone, olanzapine) or benzodiazepines is NOT an exclusion criterion and will be continued; however, patients receiving more than 2 of these substances are NOT eligible for inclusion; 6. medical history of other disorders of CNS including tics or dyskinesia; 7. medical history of cardiovascular diseases, severe hypertension, glaucoma, hyperfunction of the thyroid; 8. patients with congenital or acquired long QT syndrome, or with a familiy history of QT prolongation, sudden cardiac death or other significant inherited cardiac disorders (e.g. family history of hypertrophic cardiomyopathy); 9. history of Electroconvulsive therapy within the last 3 month; 10. known alcohol and drug addiction or abuse, except for patients with abstinence > 3 month. Patients with sporadic abuse of cannabis (products) will not be excluded from the study. That is even true with a positive THC screen in urine; 11. pregnant or nursing women; 12. concomitant participation in other clinical trials or participation during the 30 days prior to screening; 13. prior participation in this study or 14. suicidality.

### Outcome measures

#### Primary outcome measure

The primary outcome measure is the severity of manic symptoms as assessed by the Young Mania Rating Scale (YMRS) [41]. The YMRS is a clinician-administered mania rating scale. It has 11 items and is based on the patient’s subjective report of his or her clinical condition, normally over the previous 48 hours. In this study, the period is usually since the last scoring; at baseline and day 9 the scoring refers to the last 24 hours. Additional information is based upon clinical observations made during the course of the clinical interview. The items being scored are elevated mood, increased motor activity-energy, sexual interest, sleep, irritability, speech (rate and amount), language-thought disorder, content, disruptive-aggressive behaviour, appearance, and insight. The purpose of each item is to rate the severity of that abnormality in the patient. A score of ≥ 12 indicates mania. The primary endpoint is after 2.5 days of treatment.

#### Secondary outcome measures

Secondary outcome variables comprise:

• Clinical impression as assessed by the Clinical Global Impressions Scale Bipolar version (CGI–BP) [43]. This is a modified version of the Clinical Global Impressions Scale (CGI) specifically for use in assessing global illness severity and change in patients with bipolar disorder. It will be rated twice, before first and after last treatment.

• Severity of agitation as assessed by the Positive and Negative Syndrome scale - excited component (PANSS-EC) [42]. The PANSS–EC is an easy and fast to administer and easy to record scale for assessment of excitation and agitation including five items: poor impulse control, tension, hostility, uncooperativeness and excitement. It has been validated and is appropriate for repeated measures and will be scored up to 5 times per visit.

• EEG-vigilance as assessed by the ‘Vigilance Algorithm Leipzig’ (VIGALL) [44]. The VIGALL is an algorithm using topographic and spectral EEG-information for classifying different stages of wakefulness ranging from alertness over drowsiness to sleep. During stages A and B subjects are awake, during stage C asleep. Stages and sub-stages are defined as follows: stage A: high alpha power: (A1: high occipital alpha power, A2: high overall alpha power, A3: high frontal alpha power); stage B: alpha absent (B1: ‘flat’ desynchronised EEG, B2/3: high delta/theta power); stage C: K-complexes or sleep spindles. Resting EEGs will be conducted twice, before first and after last treatment.

• Total amount of movements over the study period as assessed by actigraphy.

• Cognitive performance as assessed by the “Screen for Cognitive Impairment in Psychiatry” (SCIP) [45]. The SCIP was designed for detecting cognitive deficits in several psychotic and affective disorders. It may be administered without the need for additional equipment (only pencil and paper) and requires nearly 15 min. Three alternative forms of the scale are available to facilitate repeated testing while minimising learning effects. The SCIP includes a Working Memory Test, a Verbal Learning Test-Immediate, a Verbal Fluency Test, a Verbal Learning Test-Delayed, and a Processing Speed Test. The SCIP will be performed three times, at baseline at day 1 and day 2.

### Other assessments

In addition, demographics (age, sex, ethnic origin) and vital signs (blood pressure, heart rate) will be captured. Blood analyses including haematology, clotting status and serum chemistry as well as urinalyses (dip stick and drug-screening) will be performed. Cardiac function will be monitored using a 12-lead ECG. Adverse events will be captured in the afternoon of each visit. For that, the patient will be asked a non-leading question such as ‘Have you had any health problems today/since you were last asked?’ For retrospective assessing of ADHD symptoms in childhood, the Wender Utah Rating Scale (WURS)-25 item version will be used [46].

### Randomisation

Subjects will be randomised on day -1 or 0 to either treatment arm after confirmation that the subject is eligible for the study. There will be a block randomisation. Every site will initially receive an even number of with a MedCode (e.g. 1000-1) consecutively numbered blisterpacks, half of them containing verum, the other half containing placebo. The block size ranges from 2 (alternating verum and placebo) to 6 (up to 3 times placebo/verum in a row). The number of blocks and the block size are unknown to the sites. Blisterpacks have to be used in ascending order, i.e. the first patient to be randomised receives the blisterpack with the lowest number. Immediately after randomisation (not later than 2 h), the clinical trial center of the Sponsor has to be informed about randomisation by fax.

### Statistics

#### Determination of sample size

Since there are no double-blind studies comparing treatment with methylphenidate and placebo in manic patients the following considerations have been taken into account:

1. The vigilance regulation capacity varies among manic patients: 35% of patients are expected to have a markedly instable vigilance regulation and therefore are expected to strongly respond [38]. In these cases a remission of manic symptoms after 2.5 days of treatment with methylphenidate will be operationalized as 14 points decrease in the YMRS. 35% of patients are expected to have a partially instable vigilance regulation and the expected treatment response is 7 points YMRS decrease; 30% of patients are expected to have a stable vigilance regulation and in these patients the response to treatment is expected to be 3 points YMRS decrease (placebo level);

2. Expected placebo effect after 2.5 days of treatment: 3 ± 2 points on the YMRS;

3. Expected drop out rate: 5% at maximum considering the study design (short duration, inpatients).

For the statistical testing of the primary study objective a statistical power of 1- β = 0.8, and a significance level of α = 0.05 are envisaged. Using these assumptions, the number of patients per treatment group was calculated as follows: ([z_1_ + z_2_^2^ x [σ_1_^2^ + σ_2_^2^)/(μ_1_ – μ_2_)^2^; z_1_: 0,96 (type I error of 5%); z_2_: 0,842 (power of 80%), μ_1_: mean verum group: 7.9, μ_2_: mean placebo group: 3, σ_1_: SD verum group 4.7, σ_2_: mean placebo group 2). According to this calculation, 42 patients per treatment group will be required + 5% (= 2 patients) for dropouts. Thus, 44 patients per group will be required. It is therefore planned to recruit a total of 88 patients into the study. Since the empirical basis for this power analysis is small, an adaptive interim analysis will be performed after inclusion of 20 patients per study arm with the following rules [47]:

• Early stopping due to futility, i.e. it is unlikely that the null hypothesis (H_o_) can be rejected with a larger sample size (p_1_ ≥ 0.30 (α_0_));

• Early stopping with rejection of the null hypothesis (H_o_) (p_1_ ≤ 0.03 (α_1_));

• Re-estimation of the sample size in case of continuation (if p_1_ ∈ (α_1_,α_0_)).

### Analysis

The Full Analysis Set (FAS) consists of all patients of the randomised population who receive at least one dose of treatment in either group regardless of protocol deviations. All analyses will be conducted on the FAS unless otherwise specified. Supportive analyses on the Per Protocol (PP) analysis set will be conducted for the primary outcome. The PP consists of all patients of the FAS who complete the study and do not have major protocol violations.

The primary endpoint (mean change from baseline in the YMRS total score at day 2) will be analyzed by ANCOVA. Covariables include sex, age and severity of illness at baseline. Secondary efficacy measures such as change in YMRS total score from baseline at day 2, drop-out rates, or the relationship between the stability of vigilance regulation before treatment and response to methylphenidate as well as the effects of methylphenidate on the vigilance regulation will be analyzed using descriptive statistics. The statistical hypothesis test of the primary and secondary variables will be used at a significance level of 0.05 (two-sided).

Statistical evaluations will be performed by using SPSS^®^ Version PASW 20.

## Discussion

Based upon previous reports on the benefit of psychostimulants in the treatment of mania and upon the ‘vigilance regulation model of mania’, we present here the study protocol of the ‘Methylphenidate in Mania project’ (MEMAP), a randomised double blind placebo-controlled trial to test the effectiveness of methylphenidate in the early treatment of mania.

During study protocol development, the following considerations have been made:

The screening visit on day -2 or day -1 and first treatment on day 0 accommodates both the need for an early treatment and the ICH Guideline for Good Clinical Practice requirement to provide the subject “ample time and opportunity to inquire about details of the trial and to decide whether or not to participate in the trial” [48]. In addition, it allows checking routine laboratory values and plasma levels of concomitant medication prior to first study drug administration. Furthermore, an initial placebo effect through arrival on the ward will not be considered when baseline evaluations are performed one day after arrival and not on the day of arrival. Manic symptoms will be assessed throughout 2 h after study drug intake to make sure that changes are not related to grossly different plasma levels. In addition, manic symptoms will be always assessed in the morning (12.00 at baseline, 11.00 h all other evaluations) to minimize influences of circadian mood changes.

The maximum dose of 20 mg twice daily analogous to 0.29 mg/kg (daily total dose: 40 mg ≈ 0.58 mg/kg) is lower than in the majority of 26 studies listed in a recent systematic review on the safety of therapeutic methylphenidate in adults [49] which considers methylphenidate safe in the dose range from 20 to 90 mg/day. Overall, we therefore do not expect a significantly higher frequency of side effects.

The administration of methylphenidate in addition to pre-existing mood-stabilising medication is expected to be well tolerated as this has been shown for combination therapy with other psychotropic drugs [36,37].

The short duration in this study in which patients may not receive established antimanic medication of 4.5 days at maximum (screening + treatment period), being shorter than usual wash-out or placebo run in-periods of 7 days in other studies in the same indication [50,51], justifies the treatment with placebo in the half of the patients. That is particularly true since inpatients will be closely monitored by specialised psychiatry staff allowing an immediate intervention in case of a substantial worsening of mania.

Since methylphenidate has been shown and is expected to act very rapidly [40], a treatment duration of 2.5 days has been considered sufficient.

While the study may yield the results that methylphenidate is in principle efficacious in the treatment of mania, the short study duration does not allow drawing any conclusions about efficacy and safety in the longer term what is a limitation.

In conclusion, we propose here a randomised double blind placebo-controlled study to test the efficacy and safety of methylphenidate in the early treatment of mania based upon the ‘vigilance regulation model of mania’. A positive study outcome could substantially impact our understanding of the etiopathogenesis of mania and open new perspectives in its treatment.

## Competing interests

Dr. Hegerl is an advisory board member for Lilly and Lundbeck, a consultant for Nycomed and a speaker for Bristol-Myers Squibb. Dr. Vieta has received grants and served as consultant, advisor or CME speaker for the following entities: Almirall, AstraZeneca, Bristol-Myers Squibb, Cephalon, Eli Lilly, Forest Research Institute, Gedeon Richter, Glaxo-Smith-Kline, Janssen-Cilag, Jazz, Johnson & Johnson, Lundbeck, Merck, Novartis, Organon, Otsuka, Pfizer, Pierre-Fabre, Qualigen, Roche, Sanofi-Aventis, Servier, Shering-Plough, Solvay, Takeda, Teva, the Spanish Ministry of Science and Innovation (CIBERSAM), the Seventh European Framework Programme (ENBREC), the Stanley Medical Research Institute, United Biosource Corporation, and Wyeth. Dr. González-Pinto has received grant support, acted as consultant, or given presentations for the following pharmaceutical companies: Almirall, AstraZeneca, Bristol-Myers Squibb, Otsuka, Eli Lilly, Glaxo-Smith-Kline, Janssen-Cilag, SanofiAventis, Lundbeck, Novartis, Organon, Schering-Plough, Spanish Ministry of Science and Innovation, Department of Health of the Basque Government, University of the Basque Country and Pfizer. Dr. Bauer has received Grant/Research Support from The Stanley Medical Research Institute, NARSAD and the European Commission (FP7). He was/is a consultant for AstraZeneca, Lilly, Servier, Lundbeck, Bristol-Myers Squibb and Otsuka. He has received Speaker Honoraria from AstraZeneca, Lilly, GlaxoSmithKline, Lundbeck, Bristol-Myers Squibb, Servier and Otsuka. Dr. Zimmermann has received honoraria from GSK, Pfizer, BMS, Janssen, and Servier. Dr. Edel has received speaker’s honoraria from Medice. All other authors do not report conflicts of interest related to the manuscript.

## Authors’ contributions

Dr. Kluge wrote the study protocol and the manuscript. All authors were deeply involved and contributed actively to development of both, the study protocol and the manuscript. All authors reviewed and agreed to the final version of the manuscript.

## Pre-publication history

The pre-publication history for this paper can be accessed here:

http://www.biomedcentral.com/1471-244X/13/71/prepub
